# Effect of *Pimpinellatirupatiensison* Oxidative Enzymes in STZ-induced Diabetic Rat Kidney

**Published:** 2012

**Authors:** Saddala RajeswaraReddy, Thopireddy Lavany, Ganapathi Narasimhulu, Kesireddy SathyaveluReddy

**Affiliations:** a *Division of Molecular Biology and Exercise Physiology, Department of Zoology, Sri Venkateswara University, Tirupati-517502, A.P. India.*; b *Department of Biotechnology School of Herbal Studies and Naturo Sciences Dravidian University Kuppam.*

**Keywords:** *Pimpinellatirupatiensis*, Diabetes, Mitochondrial enzymes, Oxidative stress

## Abstract

The present study was aimed to evaluate the therapeutic potential of *Pimpinellatirupatiensis*(Pt) by assaying the activities of selective mitochondrial enzymes in streptozotocin induced diabetic rats. Diabetic rats showed a significant (p < 0.01) reduction in the activities of oxidative enzymes Succinate dehydrogenase (SDH), Malate dehydrogenase (MDH), Glutamate dehydrogenase (GDH) and isocitrate dehydrogenase (ICDH). Lactate dehydrogenase (LDH) activity was significantly (p < 0.01) increased in diabetic rats. The daily oral treatment of *Pimpinellatirupatiensis*ethyl alcohol extract (750 mg/kg body weight/day) to diabetic rats for 30 days reversed the above changes in a significant (p < 0.01) manner. From our observations, we conclude that administration of Pt altered the activities of oxidative enzymes, thereby suggesting its role in mitochondrial energy production. The obtained results were compared with Glibenclamide, a standard anti diabetic drug. Thus, the modulatory effects of Pt on altering these enzymes activities afford a promise for widespread use for treatment of diabetes in the future.

## Introduction

It is projected that incidence of diabetes is on rise. Present number of diabetics worldwide is 150 million and this is likely to increase to 300 million or more by the year 2025. Reasons for this rise include increase in sedentary lifestyle, consumption of energy rich diet, obesity, higher life span, *etc*. (1). Though biguanides and sulfonylureas are valuable in treatment of diabetes mellitus, their use is restricted by their limited action, pharmacokinetic properties, secondary failure rates and accompanying side effects (2). Moreover, these therapies only partially compensate for metabolic derangements seen in diabetics and do not necessarily correct the fundamental biochemical lesion (3). Nature has been a source of medicinal treatments for thousands of years, and plants-based systems continue to play an essential role in the primary health care of 80% of the world’s underdeveloped and developing countries (4).

In insulin induced dependent diabetes mellitus (IDDM) various agents like interlukin-1 beta, interferon gamma, tumour necrosis factor alpha, alloxan and streptozotocin- could operate by forming free radicals that could attack the mitochondrial genome. (5). The increased production of free radicals in mitochondria 

may damage *β*-cells, which is known to be very sensitive to free radicals (6). Also decrease in oxygen consumption and respiratory ratio were observed by (7). Furthermore, lowering in the activities of pyruvate dehydrogenase and increase in NAD^+^/NADH ratio were reported in alloxan-induced diabetic rats (8). It has been suggested that the diabetogenicity of streptozotocin is dependent on the inhibition of the activities of citric acid cycle enzymes like isocitrate and *α*-ketoglutarate dehydrogenase (9).

In the present study we have selected a plant *Pimpinellatirupatiensis*Bal. &Subr. (Family Apiaceae; local name, kondakothimera) is a rare and endemic medicinal plant and restricted to the Seshachalam hills of the Eastern Ghats, India (10-12). Dried roots of Pt are administered along with few other ingredients to cure colic and rheumatic ailments in cattle (13). The local Yanadhi tribal community uses the tuberous roots of Pt to cure severe ulcers of stomach, throat and genital organs and also as aphrodisiac (14) and abortifacient agents (15). Fruits are used to cure asthma and are considered as an effective remedy for ‘flatulent colic’ (14).The whole plant of Pt is used to treat cough, stomach, liver problems, asthma, ulcer and tooth ache (16,17). This plant root extract is also used to treat skin disease (18) and is used as an antimicrobial agent (19) it is even given in the treatment of venereal disease and peptic ulcers (20).

There is no scientific basis for antioxidant, antidiabetic activity of Pt. There were no reports on the activity of Pt in diabetic rats with reference to carbohydrate metabolic profiles. The present work was undertaken to evaluate the effects of Pt root extract in the diabetes associated alterations in oxidative enzymes.

## Experimental

*Chemicals*

All the chemicals used in the present study were analar Grade (AR) and obtained from the following significant companies: Sigma (St.Louis, MO, USA), Fischer (Pitrsburg, PA, USA), MErk (Mumbai, India), Ranbaxy (New Delhi, India), Qualigens (Mumbai, India).

*Animals*

Wistar strain male albino rats, aged 3 months (200-250 g) were used for the present study. The total number of animals used for this study is 30. The rats were maintained on standard pellet diet and provided access to water ad libitum. They were housed in clean, dry polypropylene cages and maintained in a well ventilated animal house with 12 h light-12 h dark cycle. All the experiments were carried out between 8 am to 10 am in order to avoid circadian rhythm induced changes. 

*Induction of diabetes*

Diabetes was induced in healthy male Wistar Albino rats aged about 3 months, with body weights ranging from 200-250 g, by a single intra peritoneal injection of freshly prepared STZ (40 mg/kg b.w) dissolved in ice cold 0.1 M citrate buffer (pH 4.5) after allowing the rats for overnight fasting for 12-15 h as per the method followed by Rakieten*et al., *(21). 8 hrs after STZ administration the rats were kept for next 24 h on given 15% glucose solution to prevent hypoglycemia, as STZ is capable of producing fatal hypoglycemia due to destruction of β cells which in turn results in to massive pancreatic insulin release. Diabetes was assessed by determining the fasting blood glucose after 48 h of injection of STZ. The blood glucose levels in STZ rats were increased to markedly higher levels than normal. After a week, when the condition of diabetes was stabilized, rats with marked hyperglycemia (blood glucose level ≥ 250 mg/dL) were selected. Blood was collected from the tail vein. 

The protocol of this study was submitted to the Institutional Animal Ethics Committee and approved in its resolution No 09 (iii)/a/CPCSCA/IAEC/07-08/SVU/Zool/KSR-SRR/dated 26/6/08.

*Plant material and extraction*

Tuberous roots of *Pimpinellatirupatiensis*(Pt) were collected from Shesachalam hills, (Chittoor district, Andhra Pradesh, India) during the raining season and identified by the Taxonomist of the Herbarium, Department of Botany, S.V.University, Tirupathi. Voucher specimen (1533) was deposited in S.V.University, Tirupati, Andra Pradesh, India. These roots were air dried and powdered .The powder was stored in airtight containers and was used for the extraction. To 500 g of root powder, 1500 mL of ethyl alcohol was added the clear filtrate was evaporated to dryness under vaccum using the rotavapor at 35-40°C and further dried by freeze drying.

*Experimental design*

The rats were divided into 5 groups, six rats in each group and treated as follows:

Group I: Normal control (NC), Group II: diabetic control (DC), Group III: (D+Ea.e): Diabetic animals were treated orally with 750 mg/kg/day of Pt ethyl alcohol extract for 30 days, Group IV:(N+Ea.e): Normal animals were treated orally with 750 mg/kg b.w/day of pt ethyl alcohol extract for 30 days, Group V: (D+Glb): Diabetic animals were treated with 20 mg/kg b.w/day of Glibenclamide for 30 days.

*Analytical procedures*

After completion of 30 days treatment the animals were sacrificed by cervical dislocation and the kidney tissue was excised at 4^o^C. The tissue was washed with ice-cold saline, immersed in liquid nitrogen and immediately stored in deep freezer at -80°C for further biochemical analysis.

Estimation of Succinate dehydrogenase activity (Succinate acceptor oxidoreductase–E.C: 1.3.99.1); 10% (w/v) homogenates of the kidney tissues were prepared in ice cold 0.25 M sucrose solution and centrifuged at 1000 g for 15 min at 4°C. The supernatant fraction was used for enzyme assay. The reaction mixture in a final volume of 2 mL contained 40 μ moles of sodium succinate, and 100 μmol of phosphate buffer (pH 7.0) and 4 μmol of INT. The reaction was initiated by adding 0.2 mL of homogenate containing 20 mg of tissue as an enzyme source. The incubation was carried out for 15 min at 37°C and the reaction was stopped by the addition of 5 mL of glacial acetic acid. Zero time controls (ZTC) were maintained by addition of 5 mL of glacial acetic acid prior to the addition of the enzyme source to the incubation mixture. The formazan formed was extracted over night into 5 mL of toluene at 5°C. The color developed was measured at 495 nm in a Spectrophotometer against the toluene blank. The enzyme activity was expressed in μ moles of formazan formed/mg protein/h (22, 23)

Estimation of Malate dehydrogenase (MDH) (L-malate NAD^+^oxidoreductase–E.C: 1.1.1.37); 10% (w/v) homogenates of the Kidney tissues were prepared in ice cold 0.25 M sucrose solution and centrifuged at 1000g for 15 min at 4°C. The supernatant fraction was used for enzyme assay. The total volume 2 mL of reaction mixture contained 100 μmol of phosphate buffer (pH 7.0) 40 μmol of sodium malate, 0.1 μmol of NAD and 4 μmol of INT. The reaction was initiated by the addition of 0.2 mL of homogenate containing 20 mg of tissue as an enzyme source. The incubation was carried out at 37°C for 30 min and the reaction was arrested by adding 5 mL of glacial acetic acid. The rest of the procedure was same as described earlier for LDH. The activity was expressed in μmol of formazan formed / mg protein / h (22, 23).

Estimation of Glutamate dehydrogenase (GDH-L-Glutamate; NAD oxidoreductase – EC: 1.4.1.3); 5% (W/V) of tissue homogenates were prepared in ice cold sucrose (0.25M) solution and the contents were centrifuged at 1000 g for 15 min at 4°C. The supernatant part was used as an enzyme source. The reaction mixture in a total volume of 2 mL contained 100 μmol of phosphate buffer (pH 7.4), 40 μmol of sodium glutamate, 0.1 μmol of NAD, 2 μmol of INT and 0.2 mL containing 10 mg of tissue as an enzyme source. The reaction mixture was incubated at 37°C for 30 min. The reaction was arrested by the addition of 5 ml glacial acetic acid and the formazan formed was extracted into 5 mL of toluene. The intensity of the color was read at 495 nm against the toluene blank. The enzyme activity was expressed as μmol of formazan formed/mg protein/h (24).

Estimation of Isocitrate dehydrogenase (ICDH) (Isocitrate: NADP^+^ oxidoreductase E.C:1.1.1.42); 10% homogenates of kidney tissues was prepared in 0.25 M ice cold sucrose solution and centrifuged at 1000 g for 15 min at 4°C. The supernatant was used for the enzyme assay. The reaction mixture in a final volume of 2.0 mL contained 40 μmol of DL-isocitrate 100 μmol of magnesium chloride, 100 μmol of sodium phosphate buffer (pH 7.4), 4 μmol of INT (2-P-iodophenyl 3-P-nitrophenyl 5-phenyl tetrazolium chloride), 0.2 μmol of ADP and 0.2 μmol of NADP (for NADP^+^-ICDH).

The reaction was initiated by the addition of 0.2 mL supernatant containing 20 mg of the enzyme source and the contents were incubated at 37^o^C for 30 min. After incubation, the reaction was stopped by adding 5.0 mL of glacial acetic acid and the formazan formed was extracted overnight at 5^o^C into 5.0 mL of toluene. The colour was measured at 495 nm in a spectrophotometer against toluene blank. The enzyme activity was expressed as μmol of formazan formed/mg protein/h (25, 26).

Estimation of Lactate dehydrogenase (LDH) (L-lactate: NAD^+^ Oxidoreductase–E.C: 1.1.1.27); 10% (w/v) homogenates of the kidney tissues were prepared in ice cold 0.25 M sucrose solution and centrifuged at 1000 g for 15 min at 4^o^C. The supernatant fraction was used for enzyme assay. The reaction mixture in a final volume of 2 mL contained 40 μmol of sodium lactate, 100 μmol of phosphate buffer (pH 7.4), 0.1 μmol of NAD and 4 μmol of INT. The reaction was initiated by the addition of 0.2 mL of homogenate containing 20 mg of tissue as an enzyme source and incubated for 30 min at 37^o^C and the reaction was stopped by the addition of 5 mL of glacial acetic acid. Zero time controls (ZTC) were maintained by addition of 5 mL of glacial acetic acid prior to the addition of the enzyme source to the incubation mixture. The formazan formed was extracted over night into 5 mL of toluene at 5^0^C. The color developed was measured at 495 nm in a Spectrophotometer against the toluene blank. The enzyme activity was expressed in μmoles of formazan formed/mg protein/h (22, 23). 

*Statistical analysis*

The data has been analyzed by using SPSS (Version 13.5; SPSS Inc., Chicago, IL, USA) and M.S.Office, Exel Software for the significance of the main effects (factors), and treatments along with their interactions. The data has been compared using one-way ANOVA with Duncon’s multiple range test (DMRT) and differences were considered significant at p < 0.01.

## Results

**Figure 1.(A-E):**
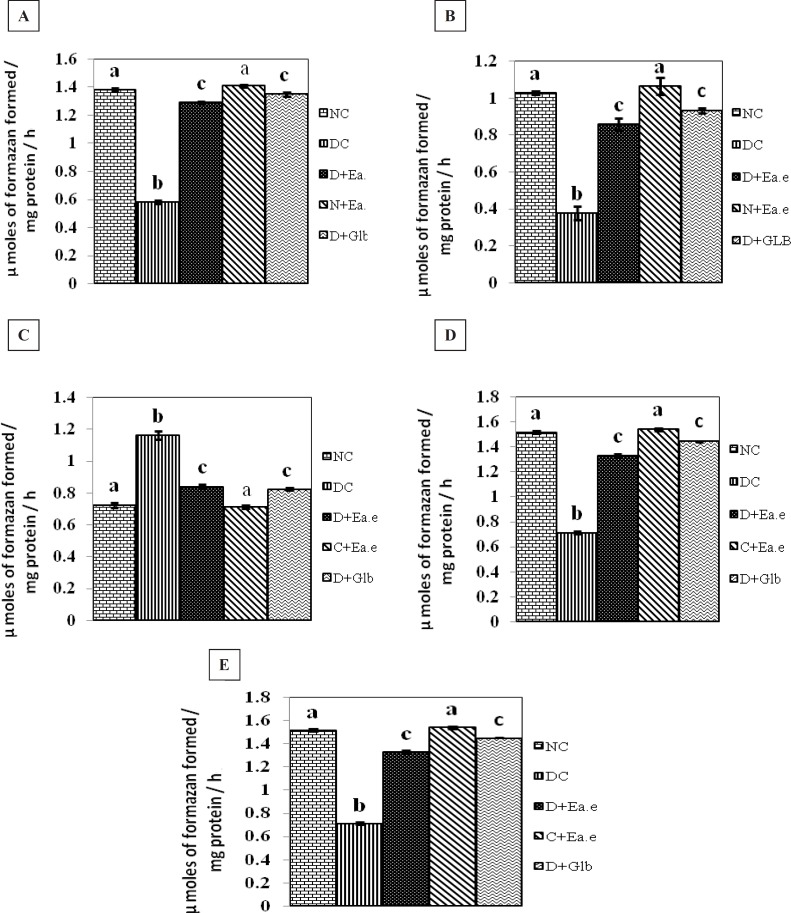
Activities of SDH, MDH, LDH, ICDH and GDH respectively in the kidney of Normal control rats (NC), Diabetic control rats (DC), Diabetic rats treated with *Pt *Ethyl alcohol extract (D+Ea.e), Normal rats treated with *Pt *Ethyl alcohol extract (N+Ea.e), Diabetic rats treated with Glibenclamide (D+Glb)*. *Each vertical bar represents the mean ± SE (n = 6). Top of the vertical bars having the same letter do not differ significantly at p < 0.01

The effect of Pt supplementation on the activities of Succinate dehydrogenase (SDH), Malate dehydrogenase (MDH), Lactate dehydrogenase (LDH), Isocitrate dehydrogenase (ICDH), Glutamate dehydrogenase (GDH), in the kidney of control and experimental groups of rats are shown in Figure 1 (A-E). There were no significant changes in the activity of these parameters in control rats treated with Pt alone. However, the activities of SDH, ICDH, GDH, MDH were decreased significantly (p < 0.01) and the activity of LDH was significantly (p < 0.01) increased in the kidney of STZ-induced diabetic rats. Treatment with Pt to diabetic groups of rats, similar to glibenclamide (GLB), the altered activities of these enzymes were regulated significantly (p < 0.01) to near normalcy in kidney.

## Discussion

In the present study, the observed decrease in the activities of mitochondrial enzymes in the kidney of the diabetic rats were significantly enhanced upon Pt treatment (Figure 1 (A-E)). In insulin dependent diabetes mellitus (IDDM) various agents like interleukin-1 beta, interferon gamma, tumor necrosis factor alpha, alloxan and streptozotocin - could operate by forming free radicals that could attack the mitochondrial genome (5). The increased production of free radicals in mitochondria may damage *β*-cells, which is known to be very sensitive to free radicals (6). Also, a decrease in oxygen consumption and respiratory ratio were observed in the mitochondria of diabetic rats (27). A similar decrease in the activities of citric acid cycle enzymes were also observed by Sener*et al., *(7). Furthermore, lowering in the activities of malate dehydrogenase and increase in NAD^+^/NADH ratio were reported by Obrosova*et al., *(8) in alloxan-induced diabetic rats. It has been suggested that the diabetogenicity of streptozotocin is dependent on the inhibition of the activities of citric acid cycle enzymes like isocitrate and *α*-ketoglutarate dehydrogenase (9). In diabetes mellitus, abnormalities of mitochondrial enzymes may impair the metabolism of glucose. As the rate of glucose oxidation normalizes insulin secretion and subsequent release of *β*-cells, a defective insulin response to glucose stimulation may be due to respiratory chain deficiency in the pancreas of IDDM. This supposes that random partitioning of mitochondria during development might have resulted in the accumulation of mutated mitochondrial DNA-containing fragments in particular tissues including pancreas. The rearrangements produce potentially antigenic chimeric proteins. The reduction in the functioning of mitochondrial enzymes may lead to a defect in the mitochondrial energy production which would impair protein synthesis and energy production in *β*-cell (28). Restoration of the activities of mitochondrial enzymes on Pt therapy suggests that it may be beneficial in enhancing protein synthesis and energy production. 

The decrease in GDH activity is attributed to its inhibition by elevated ammonia levels (product-inhibitor), which diminish the catalytic efficiency of the enzyme molecule (29). Reddy and Rao, (30) demonstrated that increased ammonia and lactate levels inhibit GDH activity. The increased LDH also reported in the present study, consonance with that lactate inhibits the GDH activity. The decrease in the activity of GDH suggests that regulation of ammonia toxicity in the kidney by the processes of deamination and amination is affected during the diabetic state. This was also reported in brain of diabetic rats by Telushkin*et al., *(31). The decrease in activities of GDH in the brain of rats with enzyme dysfunction was due to activation of lipidperoxidation (31), which attests to serious disturbances in energy metabolism and contributes to the impairment of glutamate utilization in the brain and progression of glutamate induced toxicity. The increased activity might be due to the decreased oxidative stress by Pt and increase the mitochondrial enzymes. Pt has the capacity to increase the activity of GDH in diabetic rats. There are many reports on inhibition of GDH activity by medicinal plants in diabetic rats. *Trigonella *treatment for 21 days to diabetic rats normalization of mitochondrial enzymes in diabetic rats (32).

In the current study MDH activity was decreased in diabetic rat kidney tissue. The decrease in specific activity of MDH as a consequence of diabetes suggests decreased utilization of malate. The decrease in the activity levels of dehyddrogenases is in consistent with the decreased conformation (33). An increase in proteolytic activity during diabetes may also be responsible for the decreased MDH activity. MDH activity was decreased in the tissues of diabetic animals in several studies (34, 35). The increased production of free radicals in mitochondrial cells in the tissue, also a decrease in oxygen consumption respiratory ratio were observed in mitochondria of diabetic rats. (27). Lowering in the activity of MDH and increase in NAD^+^/NADH were reported by Obrosova*et al., *(36). It has been suggested that the diabetogenecity of STZ is dependent on the inhibition of the activities of citric acid enzymes like MDH, *α*-ketoglutarate dehydrogenase (9). Diabetes decreased the expression of genes involved in carbohydrate and energy metabolism through effects on known pathways such as glycolysis, TCA cycle and oxidative phosphorylation. In diabetic rats with Pt treatment MDH levels were elevated. This may be due to decreased oxidative stress and increased activities of mitochondrial enzymes. Pt has the capacity to increase the activities of mitochondrial enzymes, this may due to the compounds which are present in Pt has the capacity to decrease the oxidative stress and increase these mitochondrial enzymes activities. There are many reports on normalization of MDH activity with medicinal plants treatment in diabetic rats. The observed decrease in the activities of mitochondrial enzymes in liver and kidney of the diabetic rats were significantly enhanced upon molybdate therapy (35). C-peptide rectified the mitochondrial defects and corrects many of the maladies associated with diabetes (37). 

SDH activity was reported to be inhibited in tissues of diabetic animals in several studies (6, 35, 38-40). The decreased activity of SDH in diabetic condition affecting succinate-fumarate conversion indicates the depressed oxidative metabolism at the level of mitochondria. A similar decrease in the activities of citric acid cycle enzymes were also observed by Sener*et al., *(7). Hyperglycemia results in decreased activities of citric acid enzymes and pentose phosphate pathways enzymes. As the phosphorylated glucose enters into the pathways like glucogenesis and glycoprotein synthesis (39, 41, 42) 

The deleterious effects of oxidative stress on mitochondrial respiration, ATP synthesis and membrane properties are mainly connected with extensive peroxidation of membranous polyunsaturated phospholipids, the integrity of which is important for functioning of mitochondrial respiratory chain. The damage of these lipids surrounding membrane bound enzymes alters the function of these enzymes (43), primarily those of mitochondrial dehydrogenases (44, 45). Long term reactive oxygen species exposure to oxidative stress resulted in oxidative damage of mitochondrial proteins that caused disturbances in mitochondrial energy production.

The decrease in SDH activity due to the STZ induced oxidative stress condition indicates reduction in the conversion of succinate to fumarate resulting in decreased in oxidative metabolism. During stress condition diversion of phosphoenolpyruvate leads to increased formation of fumarate resulting in product inhibition of SDH (46). The decrease in the activities of SDH in tissues of diabetic rats can be associated with enzyme dysfunction due to activation of lipid peroxidation. This may be due to excess production of free radicals to counter these toxic effects. In diabetic rats treated with Pt SDH activity was increased when compared to control diabetic rats. This elevation may be due to the compounds present in Pt. There are many reports on the reduction of oxidative stress by plants (35) and also plants has the capacity in normalizing the levels of lipid peroxidation. Hence by normalizing the levels of lipids the mitochondrial enzymes may become to normal level more or less in diabetic rats treated with Pt treatment. Increase in SDH activity in Pt treated rats indicates better utilization of energy yielding intermediates by TCA cycle. Same results were seen in UDCA (Ursodeoxycholic acid) treated diabetic and alcohol treated rats. This acid ameliorates the oxidative phosphorylation and normalizing mitochondrial enzymes (47). 

Lactate dehydrogenase (LDH) is a terminal glycolytic enzyme that plays an indispensable role in the interconversion of pyruvate to lactate to yield energy under anaerobic conditions (48) and the reaction occurs in both cytosolic and mitochondrial compartments (47). LDH activity is found to be altered by insulin, glucose, NADH, as well as increases in mitochondrial membrane potential, cytosolic free ATP and cytosolic free Ca^2+^ (50). The decreased activity of LDH in tissues could be important to ensure that a high proportion of both pyruvate and NADH, supplied by glycolysis, is subsequently oxidized by mitochondria. This excessive pyruvate is converted to lactate for which LDH is needed and there fore the activity of LDH may be increased due to less insulin availability in diabetes (51, 53). Increased LDH activity in diabetes has been reported by Ramachandran*et al., *(53). The results of the present study indicates the kidney LDH activity in rats with diabetes were significantly higher when compared to control. (54). Alloxan induced diabetes caused lipid peroxide mediate tissue damage in the liver, kidney, and heart (55). These changes can alter the properties and functions of the cell, resulting in either increased synthesis of some enzymes. Goldberg *et al., *(56) indicated that LDH levels were higher in patients with diabetes, than those in normal subjects. Indeed, elevated LDH levels observed in the experimental diabetic animals are associated with impaired glucose-stimulated insulin secretion (57).Thus, increased activity of LDH interferes with normal glucose metabolism and insulin secretion in the *β*-cells of pancreas and it may therefore be directly responsible for insulin secretory defects in diabetes. However, treatment with Pt to diabetic rats reverted the LDH activity to near normalcy. Similarly treatment with resveratrol to diabetic rats decreased the activity of LDH (58) most probably by regulating the proportion of pyruvate and NADH thereby promoting the mitochondrial oxidation of (pyruvate) glucose. The protective effects due to treatment with Pt strongly indicate the possibility of the extract being able to prevent any leakages of marker enzymes. There are some reports on reversal of LDH in diabetic rats with treatment with *Murray koenigii, Ocimum sanctum *(59). Stefen and Irwin (60) reported that vandate stimulates the oxidation of NADH, and hence the reduced activity of LDH in vanadyl-treated diabetic rats. 

The Isocitrate dehydrogenase (ICDH) catalyze oxidative decarboxylation of isocitrate to *α*-ketoglutarate and require either NAD^+^ or NADP^+^ producing NADH and NADPH, respectively (61). NADPH is an essential reducing equilent for the regeneration of reduced Glutathione (GSH) by Glutathione reductase and for the activity of the NADPH-dependent thioredoxin system (62, 63 ) both are important in the protection of cells from oxidative damage. Therefore, ICDH may play an antioxidant role during oxidative stress. ICDH is involved in the supply of NADPH needed for GSH production against mitochondrial and cytosolic oxidative damage (64, 65). Hence, the damage of ICDH may result in the perturbation of the balance between oxidants and antioxidants and subsequently lead to a pro-oxidant condition. We determined the activity of isocitrate dehydrogenase (ICDH) in STZ-induced diabetic rats, ICDH activity in the diabetic group was significantly lower than that in the control group. Similar results are reported by Kil*et al., *(66) they reported that mitochondrial ICDH activity was lower in diabetic group than control group.The activity of ICDH can be inhibited by glycation of ICDH. Reactive oxygen species contribute to the inactivation of ICDH by glycation. After treating with Pt the activity of ICDH was normalized, this could be due to the antioxidant activity of Pt. 

## References

[1] Yajnik CS (2001). The insulin resistance epidemic in India: fetal origins, later lifestyle, or both?. Nutr. Rev.

[2] Bailey CJ, Flatt PR, Marks V (1989). Drugs inducing hypoglycemia. Pharmacol. Ther.

[3] Taylor R, Agius L (1988). The biochemistry of diabetes. Biochem. J.

[4] King H, Aubert RE, Herman WH (1998). Global burden of diabetes, 1995–2025: prevalence, numerical estimates, and projections. Diabetes Care.

[5] Gerbitz KD (1992). Does mitochondrial DNA play a role in the pathogenesis of diabetes? Diabetologia. Diabetologia.

[6] Oexle K, Obserle J, Hibner C (1994). Insulin-dependent diabetes mellitus in melas-mitochondiropathy: discussion of possible causal relations. Modern Trends Biothermo. Kinetics.

[7] Sener A, Rasschaert J, Malaisse WJ (1990). Hexose metabolism in pancreatic islets: participation of Ca+2 sensitive to α-ketoglutarate dehydrogenase in the regulation of mitochondrial function. Biochim. Biophy. Acta.

[8] Obrosova J, Stevens MJ (1999). Effects of dietary tauric supplementation on GSH and NAD(P) redox status, lipid peroxidation, and energy metabolism in diabetic precataractous lens. Invest. Opthalmol. Vis. Sci.

[9] Boquist L, Ericsson I, Lorentzon R, Nelson L (1985). Alterations in mitochondrial aconitase activity and respiration, and in concentration of citrate in some organs of mice with experimental or genetic diabetes. FEBS Lett.

[10] Balakrishnan, P, Subramanyam, K, Bull (1960). Bot. Surv. India.

[11] Askari A, Sefidkon F, Meshkizadeh S (2004). Essential oil composition of PimpinellaeriocarpaBanks and Soland from Iran. Iranian J. Pharm. Res.

[12] Askari F, Sefidkon F (2005). Volatile components of PimpinellatragiumVill. from Iran. Iranian J. Pharm. Res.

[13] Sudarsanam G, Reddy KB, Nagaraju N (1995). Somatic embryogenesis in PimpinellatirupatiensisBal. and Subr, an endangered medicinal plant of Tirumala Hills. Int. J. Pharmacog.

[14] Thammanna, NarayanaRao K (1990). Medicinal Plants of Tirumala.

[15] Vedavathi S, Mrudula V, Sudhakar A (1997). Tribal Medicine of Chittoor District.

[16] MadhavaChetty K, Siraji K, TulasiRao K (2008). Flowering Plants of Chittor District.

[17] Rajendra Kumar Reddy P, Jayarama Reddy S (1997). Elemental concentrations in medicinally important leafy materials. Chemosphere.

[18] Jeevan Ram A, Md (2004). In-vitro antimicrobial activity of certain medicinal plants from Eastern Ghats, India, used for skin diseases. J. Ethnopharmacol.

[19] Bakshu LMD, VenkataRaju RR (2002). Essential oil composition and antimicrobial activity of tuberous roots of PimpinellatirupatiensisBal. &Subr., an endemic taxon from eastern ghats, India. Flav. Fragr. J.

[20] Nagaraju N, Rao KN, Folk-medicine for diabetes from Rayalaseema of Andhra Pradesh (1989). Ancient science of life.

[21] Rakieten N, Rakieten ML, Nadkarni MV (1963). Studies on the diabetogenic action of streptozotocin (NSC-37917). Cancer Chemother. Rep.

[22] Nachlas MM, Marguil SI, Seligman AM (1960). A colorimetric method for determination of succinate dehydrogenase activity. J. Biol. Chem.

[23] Prameelamma Y, Swami KS (1975). Glutathione dehydrogenase activity in normal and denervatedgastocnemius muscle of frog, Ranahexadactyla. Curr Sci.

[24] Lee YL, Lardy AA (1965). Influence of thyroid hormones on L-glycerophosphate dehydrogenases in various organs of the rat. J. Biol. Chem.

[25] Korenberg A, PricerWEJr (1951). Di and Triphosphate pyridine nucleotide isocitric dehydrogenase in yeast. J. Biol. Chem.

[26] Mastanaiah S, ChengalRaju D, Swami KS (1978). Circadian rhythmic activity of lipase in the scorpion, Heterometrusfulvipes(C. Koch). Curr. Sci.

[27] Puckett SW, Reddy WJ: A decrease in the malate-aspartate shuttle, glutamate translocase activity in heart mitocbondfia from alloxan-diabetic rats (1979). J Mol. Cell Cardiol.

[28] Rotig A, Bonnefont JP, Munnich A (1996). Mitochondrial diabetes mellitus. Diabetes Metab.

[29] Dudley GA, Staron RS, Murray TF, Hagerman FC, Luginbuhal A (1983). Muscle fiber composition and blood ammonia levels after intense exercise in humans. J. Appl. Physiol.

[30] Reddy MS, Rao KVR (1991). Phosphamidon, methylparathion and dichlorvas impact on tissues oxidative metabolism in penaeid prawn, Metapenaeusmonoceros. Biochem. Int.

[31] Telushkin PK, Nozdrachev AD, Potapov PP, Medvedeva NB, Stelmkh AYU (2005). Glycolysis and oxidation enzyme activiy in rat brain during insulin-induced hypoglycemia against the background of alloxan-induced diabetes mellitus Biophys. Biochem.

[32] Thakran S, Siddiqui MR, Baquer Z (2004). Trigonella foenum-graecum seed powder protects against histopathological abnormalities in tissues of diabetic rats. Mol. Cell. Biol.

[33] Cederbaum AI, Rubin E (1976). Mechanism of the protective action of cysteine and pencillamine against acetaldehyde-induced mitochondrial injury. BiochemPharmacol.

[34] Ianuzzo C, D, Armstrong RB (1976). Phosphofructokinase and succinate dehydrogenase activities of normal and diabetic rat skeletal muscle. Horm. Metab. Res. Horm. Metab. Res.

[35] Panneerselvam S, SwaminathanGovindaswamy S (2002). Effect of sodium molybdate on carbohydrate metabolizing enzymes in alloxan-induced diabetic rats. J. Nutr. Biochem.

[36] Obrosova I, Fathallah L, Lang HJ, Greene DA (1999). Evaluation of sorbitol dehydrogenase inhibitor on diabetic peripheral nerve metabolism; a preventive study. Diabetologia.

[37] Sima AA (2003). C-peptide and diabetic neuropathy. Expert. Opin. Inves. Drugs.

[38] Chen V, Ianuzzo CD (I982). Metabolic alterations in skeletal muscle of chronically streptozotocin-diabetic rats. Arch. Biochem. Biophys.

[39] Gomathy R, Vijayalekshmi NR, Kurup PA (1990). Hypoglycemic action of the pectin present in the juice of the inflorescence stalk of plantain-mechanism of action. J. Biosci.

[40] LashinOssama M, Pamela A, Szweda Luke I, Szweda Andrea MP (2006). Romani decreased complex II respiration and HNE-modified SDH subunit in diabetic heart. Free Rad. Biol. Med.

[41] Rathi AN, Aruna V, RadhaShanmugasundaram K (1981). Studies on protein-bound polysaccharide components and glycosaminoglycans in experimental diabetes-effect of GlymnemasylvestreR. Br. Ind. J. Exp. Biol.

[42] Hue L (1987). Gluconeogenesis and its regulation. Diabetes Metab. Rev.

[43] Narabayashi H, Takeshige K, Minakami S (1982). Alteration of inner membrane components and damage of electron-transfer activities of bovine heart submitochondrial particles induced by NADPH-dependent lipid peroxidation. Biochem. J.

[44] Padma G, Setty OH (1997). Effect of administration of galactosamine hydrochloride on rat liver motochondria. Indian J. Biochem. Biophys.

[45] Ramanathan k, Shila S, Kumaran S, Paneerselvam C (2003). Ascorbic acid and alpha-tocopherol as potent modulators on arsenic induced toxicity in mitochondria. J. Nutr. Biochem.

[46] Moorthy KS (1983). Modulation of Carbohydrate and Associated Metabolism in the Selected Tissues of Fresh Water Mussel, Lamellidensmarginalis During Induced Methyl Parathion Stress.

[47] Tabouy L, Zamora AJ, Oliva L, Montet AM, Beauge F, Montet JC (1998). Ursodeoxycholate protects against ethanol-induced liver mitochondrial injury. Life Sci Life Sci.

[48] Kavanagh KL, Elling RA, Wilson DK (2004). Structure of Toxoplasma gondiiLDH1: active-site differences from human lactate dehydrogenases and the structural basis for efficient APAD+ use. Biochem.

[49] Bouché C, Serdy S, Kahn CR, Goldfine AB (2004). The cellular fate of glucose and its relevance in type 2 diabetes. Endocr. Rev.

[50] Ainscow EK, Zhao C, Rutter GA (1999). Pivotal role of lactate dehydrogenase activity in the control of mitochondrial metabolism in MIN6 beta cells. Diabetologia.

[51] Awaji Y, Hastimoto H, Matsui Y, Kawaguchi K, Okumura K, Lto T, Stake T (1990). Isoenzyme profiles of creatine kinase, lactate dehydropgenase and aspartate aminotransferase in diabetic heart: comparison with hereditary and catecholamine cardiomyopathies. Cardivasc. Res.

[52] Jones RG, Grant PG, Brown D, Stickland M, Wiles PG (1988). Arise in the plasma activities of hepatic enzymes are not a common consequence of hypoglycaemia. Diabetes Med.

[53] Ramachandran B, kandaswamy M, Narayanan V, Subramanian S (2003). Insulin mimetic effects of macrocyclic binuclear oxovanadium complexes on streptozotocin-induced experimental diabetes in rats. Diabetes Obes. Metab.

[54] Zappacosta B, De Sole P, Rossi C, Marra G, Ghirlanda G, Giardina B (1995). Lactate dehydrogenase activity of platelets in diabetes mellitus. Eur. J. Clin. Chem. Biochem.

[55] Anuradha CV, Selvam R (1993). Effect of oral methionine on tissue lipid peroxidation and antioxidants in alloxan-induced diabetic rats. J. Nutr. Biochem.

[56] Goldberg DM, Martin JV, Knight AH (1977). Elevation of serum alkaline phosphate activity and related enzymes in diabetes mellitus. Clin. Biochem.

[57] Ainscow EK, Zhao C, Rutter GA (2000). Acute overexpression of lactate dehydrogenase-A perturbs beta-cell mitochondrial metabolism and insulin secretion. Diabetes.

[58] Palsamy P, Subramanian S (2009). Modulatory effects of resveratrol on attenuating the key enzymes activities of carbohydrate metabolism in streptozotocin–nicotinamide-induced diabetic rats. Chemico-Biol. Interac.

[59] Narendhirakannan RT, Subramanian S, Kandaswamy M (2006). Biochemical evaluation of antidiabetogenic properties of some commonly used Indian plants on Streptozotocin-induced diabetes in experimental rats. Clin. Exper. Pharmacol. Physiol.

[60] Stefen L, Irwin F (1987). Vandate stimulate the oxidation of NADH. Free Radic. Biol. Med.

[61] Koshland DE Jr, Walsh K, La Porte DC (1985). Sensitivity of metabolic fluxes to covalent control. Curr. Top. Cell. Regil.

[62] Chae EZ, Chung SJ, Rhee SG (1994). Thioredoxin-dependent peroxide reductase from yeast. J. Biol. Chem.

[63] Kwon SJ, Park JW, Choi WK, Kim IH, Kim K (1994). Inhibition of metal-catalyzed oxidation systems by a yeast protector protein in the presence of thioredoxin. Biochem. Biophysics. Res. Commun.

[64] Jo SH, Son Mk, Koh HJ, Lee SM, Song IH, Kim YO, Lee YS’ Jeong KS, Kim WB, Park JW, Song BJ, Huh TL (2001). Control of mitochondrial redox balance and cellular defense against oxidative damage by mitochondrial NADP+-dependent isocitrate dehydrogenase. J. Biol. Chem.

[65] Lee SM, Koh HJ, Park DC, Song BJ, Huh TL, Park JW (2002). Cytosolic NADP+-dependent isocitrate dehydrogenase status modulates oxidative damage to cells. Free Radic. Biol. Med.

[66] Kil IS, Lee JH, Shin AH, Park JW (2004). Glycation-induced inactivation of NADP+ -dependent isocitrate dehydrogenase: Implications for diabetes and aging. Free Radic. Biol. Med.

